# The Evolving Epidemiology of Elderly with Degenerative Valvular Heart Disease: The Guangzhou (China) Heart Study

**DOI:** 10.1155/2021/9982569

**Published:** 2021-04-23

**Authors:** Shangfei He, Hai Deng, Junrong Jiang, Fangzhou Liu, Hongtao Liao, Yumei Xue, Murui Zheng, Huoxing Li, Shulin Wu

**Affiliations:** ^1^School of Medicine, South China University of Technology, No. 282 of Waihuan Road, Guangzhou City, 510006 Guangdong Province, China; ^2^Department of Cardiology, Guangdong Provincial People's Hospital, Guangdong Academy of Medical Sciences, Guangdong Cardiovascular Institute, No. 96 of Dongchuan Road, Guangzhou City, 510080 Guangdong Province, China; ^3^Qinghai Province Cardio Cerebrovascular Disease Specialist Hospital, No. 7 of Zhuanchang Road, Xining City, 810012 Qinghai Province, China; ^4^Guangzhou Center for Disease Control and Prevention, No. 23 of Jiaochang Road, Guangzhou City, 510120 Guangdong Province, China

## Abstract

**Aim:**

The present study was aimed at investigating the prevalence, incidence, progression, and prognosis of degenerative valvular heart disease (DVHD) in permanent residents aged ≥65 years from Guangzhou, China.

**Methods:**

This was a prospective study based on community population. Over a 3-year span, we conducted repeated questionnaires, blood tests, and echocardiographic and electrocardiogram examinations (2018) of a random sample of initially 3538 subjects.

**Results:**

The prevalence of DVHD increased with age, average values being 30.6%, 49.2%, and 62.9% in 65-74, 75-84, and ≥85 years of age, respectively. The incidence rate was 1.7%/year. Aortic stenosis was the result of DVHD, and the mean transvalvular pressure gradient increased by 5.6 mmHg/year. The increase of mild aortic stenosis was lower than that of more severe disease, showing a nonlinear development of gradient, but with great individual variations. Mortality was significantly increased in the DVHD group (HR = 2.49). Risk factors for higher mortality included age (*χ*^2^ = 1.9, *P* < 0.05), renal insufficiency (*χ*^2^ = 12.5, *P* < 0.01), atrial fibrillation (*χ*^2^ = 12.2, *P* < 0.01), mitral regurgitation (*χ*^2^ = 1.8, *P* < 0.05), and tricuspid regurgitation (*χ*^2^ = 6.7, *P* < 0.05) in a DVHD population.

**Conclusions:**

DVHD was highly prevalent among residents in southern China. With the progression of the disease, the mean transvalvular pressure gradient accelerated. DVHD was an independent predictor of death, and the mortality was higher in those with older age, renal insufficiency, atrial fibrillation, mitral regurgitation, and tricuspid regurgitation.

## 1. Introduction

Degenerative valvular heart disease (DVHD), characterized macroscopically as increased leaflet thickness, stiffening, and calcification, without commissural fusion, is a common heart disease among the elderly [1]. In the Euro Heart Survey, degenerative valvular calcification was found by echocardiography in 63% of cases [2]. Valvular calcification causes stenosis or regurgitation of one or more heart valves, resulting in abnormal heart function and eventually leading to heart failure [3]. DVHD progresses over years with a long asymptomatic phase, with symptoms occurring only at an advanced stage of disease [4]. The detection of cardiac murmur enables early diagnosis [5] but lacks specificity. Echocardiography is the preferred method to confirm the diagnosis of DVHD and to evaluate its severity [5, 6]. Severity of DVHD leads to decreased coronary blood flow, cerebral dysfunction, syncope, angina pectoris, and even death [4].

Much of the current knowledge concerning DVHD is based on hospital studies. Due to the long and asymptomatic progression of the disease, these data can never give a full picture of the prevalence, progression, and prognosis of DVHD. Large-scale community-based epidemiological surveys of DVHD are particularly rare in developing countries, especially in China. The Guangzhou (China) Heart Study was a prospective community population-based study that includes complete questionnaires, blood tests, and echocardiographic and electrocardiogram data, and was aimed at investigating the prevalence, incidence, progression, and prognosis of DVHD in permanent residents aged ≥65 years from Guangzhou, China.

## 2. Methods

### 2.1. Study Population

**Figure 1:**
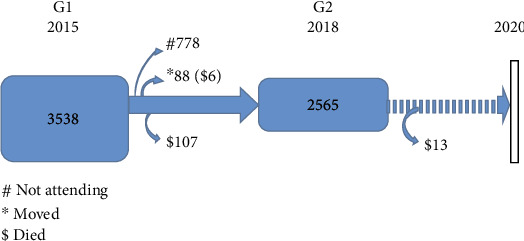
Flow chart of the cohort in the Guangzhou (China) Heart Study. The G1 Study consists of 3538 subjects. 2565 subjects participated repeatedly in the G2 Study. During follow-up, 88 had moved/emigrated and 126 had died. There were 778 subjects not attending.

The Guangzhou (China) Heart Study was initiated in 2015 and was an ongoing population-based cohort study in the community of Guangzhou. Guangzhou is a modern city in southern China with 11 districts (Yuexiu, Haizhu, Liwan, Tianhe, Huangpu, Baiyun, Panyu, Nansha, Huadu, Conghua, and Zengchen districts). We started the Guangzhou (China) Heart Study in 2015 (G1 Study). A 5-stage, stratified multistage random sampling method was used to recruit subjects [7]. Initially, all the 11 districts were divided into an urban group and a rural group, from which one urban district and one rural district were selected by a simple random sampling method. Later, the second stage of sample selection consisted of streets or townships and this stage leads to selecting streets or townships. The third stage of sample selection consisted of residential committees or village committees, and this stage leads to selecting residential or village committees. The fourth stage of sample selection consisted of households; this stage leads to selecting households. Finally, the fifth stage leads to selecting subjects within the selected households. The inclusion criteria were as follows: (1) permanent residents (resident in Guangzhou for at least 6 months) and (2) ≥65 years of age. The exclusion criteria were residents with mental or cognitive disorders, disturbance of understanding, deaf-mutters, mobility difficulties, and malignant tumors. Eventually, the G1 Study consists of 3538 subjects. Over a 3-year span, we conducted the Guangzhou (China) Heart Study again (G2 Study). 2565 subjects participated repeatedly, with a return visit rate of approximately 75%. During follow-up, 88 had moved/emigrated and 126 had died. There were 778 subjects not attending (Figure 1).

### 2.2. Transthoracic Echocardiography Protocol

All subjects underwent echocardiographic examinations, which was performed using a Vivid i cardiovascular ultrasound system (GE Healthcare, Horten, Norway, Probe Frequency 2.5 MHz). The standard parasternal long-axis, short-axis, suprasternal, subcostal, and apical four-chamber views were performed on each subject [8]. In order to ensure the image quality, all echocardiograms were judged and confirmed by two ultrasound experts with more than 10 years of working experience. Poor echocardiograms were excluded. Degenerative mitral valve calcification is a form of valvular calcification that occurs at the fibrous base of the mitral valve [9]. Degenerative aortic valve calcification is characterized by valvular calcification on the cusps of the aortic valve and ranges from aortic sclerosis to aortic stenosis (AS) [9]. Severity of AS was graded as mild (mean transaortic gradient (MG) 15-25 mmHg or velocity 2-2.9 m/sec), moderate (MG 25-40 mmHg or velocity 3-3.9 m/sec), or severe (MG > 40 mmHg or velocity > 4 m/sec) [10]. Mitral regurgitation (MR), aortic regurgitation (AR), and tricuspid regurgitation (TR) were graded qualitatively by Doppler color flow imaging, and severe valvular regurgitation was defined as follows: (1) severe MR with a grade ≥ 3/4; (2) severe AR with a grade ≥ 3/4; and (3) severe TR with a grade ≥ 3/4 [11].

### 2.3. Cohort Definition

Hypertension was defined as a blood pressure > 140/90 mmHg or a history of hypertension and the use of antihypertensive medications. Diabetes was defined as a level of fasting blood glucose ≥ 7.0 mmol/L or taking antidiabetic medications. Chronic kidney disease (CKD**)** was defined as serum creatinine level ≥ 2 mg/dL [11]. Atrial fibrillation (AF**)** was diagnosed when standard 12-lead electrocardiogram or 24-hour single-lead electrocardiogram (≥30 s) showed intermittent P wave and irregular RR interval (atrioventricular conduction was not impaired) [12].

### 2.4. Ethics

The written informed consent was obtained from all participants. This study was approved by the Guangzhou Medical Ethics Committee of the Chinese Medical Association (No. GDREC2015306H) and was conducted in accordance with the ethical standards of the 1964 Helsinki Declaration and its later amendments or comparable ethical standards.

### 2.5. Statistical Analysis

We performed prevalence calculations, first as the point prevalence associated with the G1 and G2 Study and as the weighted mean of the combination of two studies (G1/2). The study population was divided into three age cohorts. Group comparisons were made using Student's *t*-test for continuous variables and the *χ*^2^ test for categorical variables. We used the following equation to calculate incidence rate: incidence rate/year = *X*/(*N*–1/2*C*–1/2*X*), where *X* is the number of incident cases with DVHD, *N* is the number in the study population, and *C* is the censored participants (censoring occurred when participants moved or at the end of the study, not as a result of nonattendance). Survival analysis was conducted using various statistical tools, such as Cox regression models and Kaplan-Meier analysis. Hazard ratio (HR) and 95% confidence interval (CI) were calculated to assess the associations. A two-sided *P* < 0.05 was considered to be statistically significant. Statistical tests were performed using SPSS (version 20.0; SPSS, Inc., Chicago, IL, USA).

## 3. Results

### 3.1. Baseline Characteristics

As shown in Table 1, 3538 subjects were included, and the mean age was 72.0 ± 5.8 years of age. 61.9% were female. Diabetes mellitus accounted for 15.1%, hypertension in 50.5%, current smoking in 23.2%, AF in 4.6%, and CKD in 49.1%. Most of them had valvular regurgitation; AR was present in 67.5%, TR in 67.4%, and MR in 60.7%. 1355 subjects were diagnosed with DVHD; the mean age was 73.9 ± 6.2 years of age. Degenerative aortic valvular calcification was 37.2%. In addition, degenerative mitral valvular calcification was present in 8.2%.

### 3.2. Prevalence

As shown in Table 2, the prevalence of DVHD increased with age (*P* < 0.01), weighted mean values of G1/2 being 30.6% (95% CI; 28.2%-32.9%) in 65-74 years of age, 49.2% (95% CI; 43.3%-49.8%) in 75-84 years of age, and 62.9% (95% CI; 52.3%-67.0%) in ≥85 years of age. There were gender differences in the prevalence of the aged 65-74 group in the two studies, with more males than females, which could be explained by differences in age distribution between male and female.

### 3.3. Incidence

There were 3538 subjects in the G1 Study. Over a 3-year span, we conducted the G2 Study and 2565 subjects participated repeatedly. 42 subjects with DVHD were newly diagnosed by echocardiography. Therefore, we defined 42 subjects as incident cases in the G2 Study. Then, the incidence rate was calculated using the formula described in Statistical Analysis, and the result was 1.7%/year (95%CI ± 0.65%).

### 3.4. Progression

AS was the result of DVHD. There were 32 subjects with aortic stenosis in the G1 Study. Over a 3-year span, we conducted the G2 Study and 32 subjects participated repeatedly. All subjects had two measurements of MG. The mean progression/year was 5.6 mmHg, with a wide SD of 6.0 and a range from -2.9 to 28.3. The progression rate in subjects with an initial gradient ≥ 30 mmHg was 8.9 mmHg/year, exceeding the rate of 5.3 mmHg/year in those with a gradient < 30 mmHg (*P* < 0.05). The increase of mild AS was lower than that of more severe disease, showing a nonlinear development of gradient, but with great individual variations.

### 3.5. Mortality

**Figure 2:**
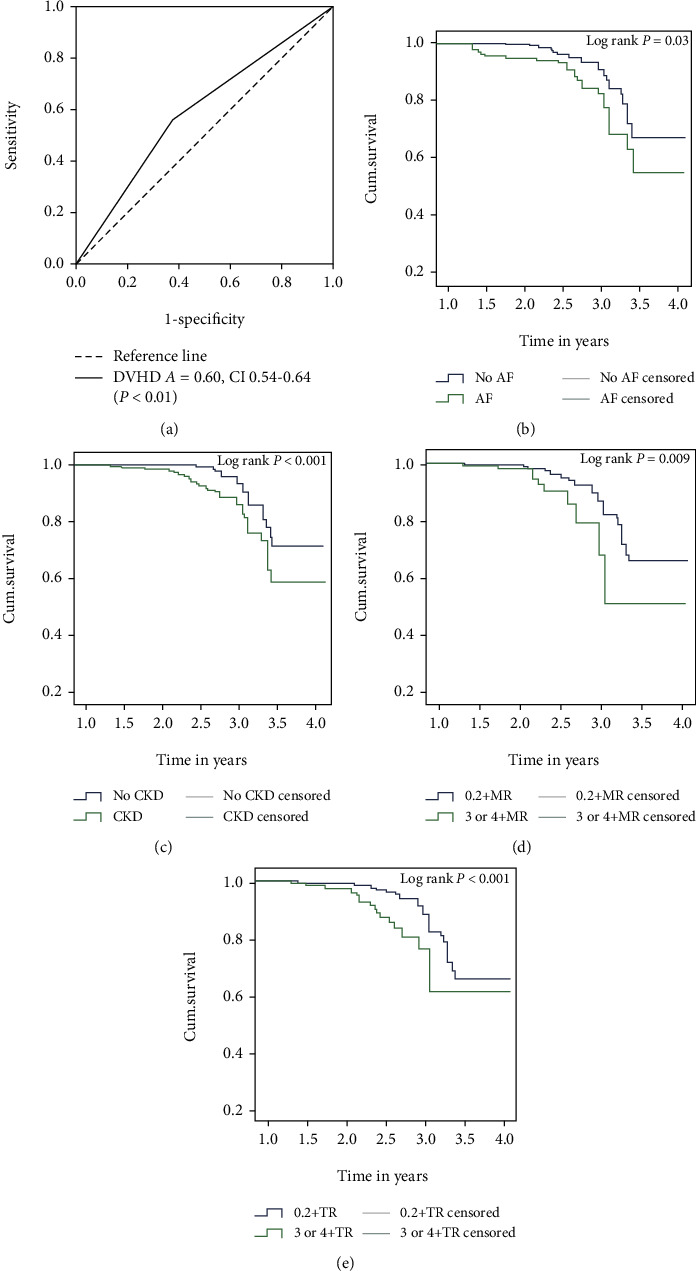
(a) ROC curve for determination of predictive value of DVHD for death. Kaplan-Meier survival curves of subjects with DVHD as presence or absence of atrial fibrillation, chronic kidney disease, severe mitral regurgitation, and tricuspid regurgitation: (b) atrial fibrillation, (c) chronic kidney disease, (d) mitral regurgitation, and (e) tricuspid regurgitation.

During the follow-up period, 126 subjects (3.6%) died until 2020. The Cox regression model was used to confirm whether DVHD was a risk factor for all-cause mortality. After adjusting for age, DVHD (HR 2.49, 95% CI 1.72 to 3.62) were strongly associated with risk for death. Receiver operating characteristic (ROC) curves were generated for DVHD to determine its diagnostic capability for death. The area under the ROC curve (AUC) for DVHD was 0.60 (95% CI 0.54 to 0.64) (Figure 2(a)).

We performed Kaplan-Meier analysis to evaluate the risk factors for all-cause mortality in a DVHD population. Univariate clinical associations of poor survival included older age (*P* < 0.01), AF (*P* < 0.01), and CKD (*P* < 0.01) (Figures 2(b) and 2(c)). These were independent clinical factors associated with survival after adjusting for gender, current smoking, hypertension, and diabetes mellitus. Gender, current smoking, hypertension, and diabetes mellitus were not associated with survival. Among the echocardiographic variables, severe MR and TR (*P* < 0.01; Figures 2(d) and 2(e)) were associated with lower survival. AR and AS were not associated with survival. All these individual variables were tested after adjusting for all the clinical variables.

As shown in Table 3, a comprehensive multivariate survival model was established to provide approximate contributions of various factors to all-cause mortality. First, we established a stepwise regression model of survival that included all clinical variables, including age, gender, current smoking, hypertension, diabetes mellitus, CKD, and AF. Age (*χ*^2^ = 1.9, *P* = 0.019), CKD (*χ*^2^ = 12.5, *P* < 0.001), and AF (*χ*^2^ = 12.2, *P* < 0.001) were significant independent predictors of survival in this model, with a global Wald *χ*^2^ statistic of 26.5. The final stepwise model adds echocardiographic variables, including MR, TR, AR, and AS. MR (*χ*^2^ = 1.8, *P* = 0.033) and TR (*χ*^2^ = 6.7, *P* = 0.016) were significant independent predictors of survival in this model, with a global Wald *χ*^2^ statistic of 46.6.

## 4. Discussion

There were several main findings in our survey. First, DVHD was the most common valvular heart disease among the elderly. Second, it revealed an accelerated progression of the aortic mean gradient with the development of the disease. Third, DVHD was an independent predictor of death and the mortality was higher in those with older age, CKD, AF, MR, and TR.

### 4.1. DVHD Prevalence

VHD is a common heart disease among the elderly [13]. Previous studies have shown that the most common etiology is rheumatic fever in developing countries, while degenerative etiology is the main etiology in developed countries [2, 13]. Calcified aortic valve disease afflicts between 2.3 and 51.7% of elderly; with the aging of the population, the prevalence of the disease has increased significantly [13]. Mitral annular calcification is a less common form of valvular calcification that occurs at the fibrous base of the mitral valve. It affects 9-20% of the population [14]. Most data on the prevalence of VHD in developing countries comes from Asia. In a survey in Turkey that included 1300 patients hospitalized in 42 centers, rheumatic VHD accounted for 46% of subjects [15]. Due to the superior social and economic status and favorable living environment found in China, the etiology of VHD may have changed. A retrospective hospital-based survey in China suggested that rheumatic fever and degenerative valvular changes remained predominant etiologies in patients < 65 and ≥65 years of age, respectively [16]. In our survey, degenerative diseases had become the leading cause of VHD in a population ≥ 65 years of age, average values being 30.6%, 49.2%, and 62.9% in 65-74, 75-84, and ≥85 years of age, respectively, which were consistent with the data from previous cross-sectional population studies [2]. DVHD progresses over years with a long asymptomatic phase. The wide time span enables us to track this slowly progressive disease, adding knowledge to the overall incidence of the disease. From the results of the G1-G2 Study, we calculated that the incidence rate of DVHD was 1.7%/year. With the aging of the population, the DVHD population has increased significantly. Therefore, our results reflect the real situation of DVHD.

Calcified aortic valve disease was the most common cause of AS [16]. Previous prospective natural history studies of patients with AS have shown that average transvalvular pressure gradient increased by 7 mmHg/year [17]. The latest medical trial data from statins in the treatment of mild/moderate AS show that the rate of disease progression slows down as the average transvalvular pressure gradient increases for 3-4 mmHg/year [18]. These data are consistent with our results, indicating that the average transvalvular pressure gradient increased by 5.6 mmHg/year. In addition, our progression analysis reveals a nonlinear development of the disease, which becomes more rapid with the increase of average gradient. This phenomenon can be explained by assuming that the calcification process is constant over a period of time. The effect of a given narrowing of an already small valve area on the gradient is greater than that of only slight/moderate area reduction of valve. Regardless of the initial gradient, subjects show significant individual differences in disease progression. Our data suggest that the past progression rate should be taken as a factor when considering the future visit interval of AS population.

### 4.2. High Mortality and Relative Risk Factors

Previous studies have shown that all-cause mortality and cardiovascular death increased significantly in calcified aortic valve disease patients during the 5-year follow-up period; even after adjusting for age, gender, and baseline factors, calcified aortic valve disease is still associated with higher mortality and cardiovascular risk [19]. Similarly, all-cause mortality and cardiovascular mortality in patients with mitral valve calcification increased by 53% and 65%, respectively, compared with patients without mitral valve calcification, similar to what Coffey and colleagues found in another meta-analysis [13]. In summary, the presence of valvular calcification is a major cause of mortality and an increase in major cardiovascular events, which have been reported in the Framingham Study [20]. Rossi et al. [21] described the relationship between valvular calcification and all-cause mortality; these associations were not related to diabetes mellitus, CKD, and echocardiographic variables (left ventricular mass, left ventricular ejection fraction, and left atrial diameter). These data are consistent with our results, indicating that DVHD may be the direct cause of increased mortality.

In our present study, the comprehensive multivariate survival model showed that the DVHD population with older age, CKD, AF, MR, and TR had a higher risk of death. The effects of age and CKD were not surprising. AF was associated with age and valvular regurgitation but was associated with higher mortality independent of these factors [22]. Left atrial (LA) enlargement has always been considered to be the result of MR. [23] Several pieces of evidence suggest that marked LA enlargement may predict arrhythmias and mortality [24] and may independently and gradually predict the severity of MR. In conclusion, LA enlargement has a strong and independent relationship with excessive mortality in degenerative MR. The significant variability of LA response to degenerative MR is associated with subsequent survival, which has a considerable impact on degenerative MR management. Most TR are classified as functional or associated with pulmonary hypertension, right ventricular dysfunction, or both. Some evidence suggests that severe TR has independent relationship with lower survival [25]. Right atrial enlargement has always been considered to be the result of TR. After adjusting for possible confounders, severe TR was associated with a 3-fold increase in the risk of 1-year mortality. Similarly, after multivariate adjustment, larger right atrium size and right ventricular dilation were associated with increased mortality [26].

Degenerative disease is the most frequent cause of AR and is characterized by the combination of enlargement of the aortic root and abnormalities in the morphology and mobility of aortic leaflets [27]. We did not observe an association between AR and mortality, while several other studies have demonstrated an association between AR and lower survival [28]. However, in some of these studies, the association between AR and lower survival was quite modest. For example, Badiani et al. recently reported that mild to moderate AR was not associated with clinical events (death, aortic valve replacement, and cardiac hospitalization) [29]. While patients with severe AR was associated with increased long-term postoperative mortality, this association was quite modest (HR = 1.81) [30]. Therefore, we believe our finding that AR were not associated with mortality was not an anomaly. This may be explained by several factors. First of all, the relationship between AR and mortality may not be linear and may be more clearly observed when grouping patients based on the severity of AR. Second, in our survey, the relationship between severe AR and mortality is diluted by a large number of mild to moderate AR of cases. This would confound a clear association between AR and increased mortality.

## 5. Study Limitations

The study had some limitations. First of all, our survey included only the community population aged ≥65 years in Guangzhou, which was neither nationally representative nor ethnically diverse; the results may underestimate the actual prevalence of DVHD in China. Second, not everyone was able to participate in all screening programs in fully voluntary population screening or for reasons such as disease and death.

## 6. Conclusions

This study described the epidemiological characteristics of the evolution of DVHD. DVHD was the most common valvular heart disease among the elderly. It revealed an accelerated progression of the aortic mean gradient with the development of the disease. DVHD was an independent predictor of death, and older age, AF, CKD, MR, and TR are associated with subsequent survival, which have a considerable impact on the management of DVHD.

## Figures and Tables

**Table 1:** Characteristics for the total cohort and the subgroup having DVHD.

Variable	G1^¶^	G2
Total population	3538	2565
Mean age	72.0 ± 5.8	74.5 ± 5.6
Male gender, *n* (%)	1348 (38.1)	957 (37.3)
Female gender, *n* (%)	2190 (61.9)	1608 (62.7)
Hypertension, *n* (%)	1788 (50.5)	1502 (58.6)
Diabetes mellitus, *n* (%)	534 (15.1)	463 (18.1)
Atrial fibrillation, *n* (%)	163 (4.6)	138 (5.4)
Renal insufficiency, *n* (%)	1736 (49.1)	1346 (52.5)
Current smoking, *n* (%)	822 (23.2)	594 (23.2)
Serum creatinine level (mg/dL)	2.16 ± 1.3	2.14 ± 1.0
DVHD^†^ population	1355 (38.3)	953 (37.2)
Mean age	73.9 ± 6.2	76.1 ± 6.1
Male gender, *n* (%)	549 (15.5)	386 (15.0)
Female gender, *n* (%)	806 (22.8)	567 (22.1)
DVHD classification		
DAVC^‡^, *n* (%)	1317 (37.2)	926 (36.1)
DMVC^§^, *n* (%)	289 (8.2)	244 (9.5)
Mild aortic regurgitation, *n* (%)	1856 (52.5)	1369 (53.3)
Moderate-severe aortic regurgitation, *n* (%)	531 (15.0)	411 (16.0)
Mild mitral regurgitation, *n* (%)	1867 (52.8)	1352 (52.7)
Moderate-severe mitral regurgitation, *n* (%)	282 (8.0)	221 (8.6)
Mild tricuspid regurgitation, *n* (%)	1915 (54.1)	1378 (53.7)
Moderate-severe tricuspid regurgitation, *n* (%)	470 (13.3)	366 (14.3)
Aortic stenosis, *n* (%)	39 (1.1)	27 (1.1)

Data are expressed as mean ± SD or as percentages. ^**†**^Degenerative valvular heart disease; ^‡^degenerative aortic valvular calcification; ^§^degenerative mitral valvular calcification; ^¶^Guangzhou Heart Study.

**Table 2:** Prevalence of DVHD for three age groups in G1 and G2 and also as a weighted mean values of all two surveys.

	Prevalence (%)		
Age cohort	G1^¶^	G2	G1/2
65-74 years	31.1	30.0	30.6
Male	34.6^∗∗∗^	34.0^∗∗∗^	
Female	29.2	27.9	
75-84 years	53.8	44.6	49.2^∗^
Male	53.0	46.6	
Female	54.5	43.5	
≥85 years	65.9	59.9	62.9^∗∗^
Male	66.7	59.4	
Female	65.3	60.2	

Guangzhou Heart Study. Prevalence numbers (%) are calculated for three age cohorts in G1 Study and G2 Study and also as a weighted mean of all two surveys; ^∗^*P* < 0.01 between 75-84-year and 65-74-year groups; ^∗∗^*P* < 0.01 between ≥85-year and 75-84-year groups; ^∗∗∗^*P* < 0.05 between male and female.

**Table 3:** Comprehensive multivariate model to predict survival in subjects with DVHD.

Variable	Hazard ratio (95% CI)	*P*	Wald *χ*^2^
Age per 10 years	1.11 (1.06-1.17)	0.019	1.9
Current smoking	1.08 (0.63-1.84)	0.793	1.4
Hypertension	0.96 (0.58-1.58)	0.872	1.1
Diabetes mellitus	1.83 (0.77-2.31)	0.313	1.0
Chronic kidney disease	2.74 (1.57-4.80)	<0.001	12.5
Atrial fibrillation	2.47 (1.49-4.11)	<0.001	12.2
All clinical variables	—	<0.001	26.5
Mitral regurgitation per grade	1.10 (1.02-1.18)	0.033	1.8
Tricuspid regurgitation per grade	2.69 (1.21-6.00)	0.016	6.7
Aortic stenosis per grade	1.03 (0.22-4.83)	0.968	1.1
Aortic regurgitation per grade	1.36 (0.70-2.64)	0.363	1.7
Clinical + echocardiographic	—	<0.001	46.6

## Data Availability

The data used to support the findings of this study are available from the corresponding author upon reasonable request.
